# Editorial: The role of nutritional strategies in the regulation of gut microbiota and host immune system

**DOI:** 10.3389/fmicb.2024.1501596

**Published:** 2024-12-05

**Authors:** Li Wang, Caihong Hu, Ming Z. Fan

**Affiliations:** ^1^State Key Laboratory of Swine and Poultry Breeding Industry, Institute of Animal Science, Guangdong Academy of Agricultural Sciences, Guangzhou, China; ^2^Key Laboratory of Molecular Animal Nutrition, Ministry of Education, College of Animal Science, Zhejiang University, Hangzhou, China; ^3^Department of Animal Biosciences, Ontario Agricultural College, One Health Institute, University of Guelph, Guelph, ON, Canada

**Keywords:** gut microbiota, host immune, intestinal inflammation, intestinal barrier, nutritional strategies

Gut microbes refer to the microbial communities that live in the gut of animals and humans, including bacteria, fungi, viruses and other microorganisms. These microbes form complex symbiotic relationships to their hosts with important effects on performances and productivity, health and disease status of their hosts (Kuziel and Rakoff-Nahoum, 2022). There are close interactions between the gut microbiota and the host immune system. Studies have shown that the gut microbiota help to maintain intestinal homeostasis by regulating the host development and function of the immune system (Yang et al.). The topic of gut microbiota is of a significant concern, and according to the Web of Science, using the keyword gut microbiota, approximately 111,936 articles have been so far published *per se* on this topic, thus how gut microbiota affect host overall physiological status and health is important. Under this context, this special Edition topic has published a total of 25 articles including two reviews and one opionion, two disease related studies, seven poultry research, seven herbivore studies, three pig studies, two neonatal calf and piglet studies and one wildlife animal research.

The gut microbiota not only play important roles in many metabolic pathways, but also provide the host with a variety of physiological functions (Zhu et al.). Intestinal flora, on the one hand, they stimulate the development and maturation of the host immune system, activate the relevant lymphoid tissues, enhance immunoglobulin A (IgA) secretion, improve immune recognition, and induce cytokine production by Giant cells and T and B lymphocytes; and on the other hand, they regulate hosts' immune responses by regulating the strength of the immune response and avoiding tissue damage caused by excessive immune response (Yang et al.). Gut flora play a key role in maintaining host immune homeostasis. Through competitive inhibition and the production of antimicrobial substances, the intestinal flora are able to resist the invasion of foreign pathogens and protect the host from infection (Yang et al.). Through bioinformatic analyses, the disease and health-genetic causal relationship between the gut microbiome and psoriatic arthritis was observed (Qiann et al.). At the same time, intestinal flora can also regulate the distribution and functional status of hosts' immune cells to ensure the normal operation of their immune systems. The gut microbiota and their metabolites and components are essential for immune homeostasis and influence hosts' susceptibility to various immune-mediated diseases and conditions (Yang et al.; Zhu et al.). For example, intestinal flora can produce various microbial metabolites such as short-chain fatty acids (SCFA), tryptophan metabolites secondary bile acids, and other metabolites, which are able to regulate the immune response through the activation of specific neurons and immune cells, thus maintaining the immune homeostasis of the intestinal mucosa (Yang et al.). The composition of the gut microbiota influences the development of the immune system and regulates immune mediators, which in turn influences the function of the gut barrier.

The gut microbiome sends information to the body about inchoate environmental exposures, such as dietary components and allergens, promotes tolerance to these environmental factors, helps the immune system recognize commensal bacteria and inhibits pathogenic bacterial proliferation. Numerous studies have shown that the primitive development of the immune system requires the cooperation of the gut microbiota, such as promoting the development of the primary immune organs of spleen and thymus, increasing the number of immune cells in lamina propria, and promoting the production of IgA in intestinal mucus. Tian et al. (2023) reported that antibiotic exposure in early life was associated with reduced gut microbiota diversity and abundance in adulthood. Hepatocyte interaction networks influenced by the gut-liver axis play a critical role in regulating liver-resident natural killer cell maturation and function, which may be the key to the early development of the immune system (Tian et al., 2023). Early life is also a critical time for the interaction between gut microbiota and host immunity; and early life is a critical period for the development of intestinal flora and a critical window period for the maturation of the immune system (Tian et al., 2023). Thus, development of the immune system requires the cooperation of intestinal microorganisms to maintain homeostasis of the gut microbiota and to reduce susceptibility to a variety of immune diseases.

Composition and balance of the gut microbiota are closely related to the nutritional and physiological functions of the host. In the past several decades, the adoption of the modern industrialized dietary habits has become a growing health concern, as it is strongly associated with obesity and related metabolic diseases, promoting inflammation and both structural and behavioral changes in gut microbiota. In this context, novel dietary strategies are emerging to prevent diseases and maintain health (Tian et al., 2023). However, the consequences of these different diets on gut microbiome modulation are still largely unknown, and could potentially lead to alterations of gut microbiome, intestinal barrier, and the immune system. Panah et al. investigated the effect of a westernized diet and concluded that a westernized diet could be a potential onset risk factor and an exacerbating agent for chemically inducing ulcerative colitis with dextran sulfate sodium in the pig model by reducing abundances of the SCFA-producing bacteria, increasing the abundance of pathogens such as *Helicobacter trogontum*, and by increasing the concentration of microbial proteolytic-derived metabolites in the colon. Earlier studies suggested that Mediterranean diets (MedDiet) might have anti-inflammatory effects (Casas et al., 2018). MedDiet mainly provide long-chain polyunsaturated omega-3 fatty acids from fish and nuts, polyphenols from red wine and fruits and probiotics from yogurt products (Chiavaroli et al., 2018). Research data further illustrate that the MedDiet reduce the incidence of cardiovascular diseases by 28% (Chiavaroli et al., 2018; Zhu et al.).

Gut microbial disorders can disrupt intestinal-brain homeostasis and tight junctions in influencing metabolism, immunity and endocrine systems. The resulting increase in intestinal permeability and formation of a “leaky gut” can increase the infiltration of large amounts of pathogenic bacteria and toxic metabolites into the bloodstream, causing local or systemic inflammation (Obrenovich, 2018). The inflammatory response can also disrupt the blood-brain barrier, known as the leaky brain, and substances that promote inflammation, such as lipopolysaccharides (LPS), may enter the brain, leading to neuroinflammation, mental disorders, and abnormal behavior (Obrenovich, 2018; Yousefi et al., 2022). On the other hand, probiotics have been shown to exert beneficial effects on host health and behavior, including reversing stress or antibiotic-induced gut microbial disorders and restoring physiological and behavioral changes in the host by modulating the gut-brain axis signaling via hormones, immune factors, etc. (Arslanova et al., 2021; Wang P. et al., 2021). The effects of dietary supplementation of *Bacillus subtilis DSM29784* (BS) on the prevention of subclinical necrotic enteritis (SNE) were evaluated in broilers; and it was indicated that the BS pretreatment increased villus height, claudin-1 expression, maltase activity, and immunoglobulin abundance, while decreasing lesional scores, as well as mucosal IFN-γ and TNF-α concentrations (Wang Y. et al.). In addition, the BS pretreatment increased the relative abundance of beneficial bacteria and decreased that of pathogenic bacteria (Wang Y. et al.). The effects of dietary supplementation of the probiotic *Lactobacillus rhamnosus LR-32* on the gut microbiota, barrier integrity, and 5-hydroxytryptamine (5-HT) or serotonin metabolism, it was found that *Lactobacillus rhamnosus LR-32* supplementation restored the profile of the gut microbial community, and showed positive effects by increasing the expression of tight junction proteins in the ileum and hypothalamus and promoting the expression of genes related to central 5-HT metabolism laying hens (Huang C. et al.). In addition, Han et al. investigated the effects of dietary supplementation of 10-hydroxy-2-decenoic acid (10-HDA) on the growth performance, intestinal barrier, inflammatory response, oxidative stress, and gut microbiota of broiler chickens challenged with LPS, and found that dietary 10-HDA supplementation alleviated the LPS-induced intestinal mucosal injury and the loss of growth performance through anti-inflammatory, antioxidant, and gut microbiota modulation activities. Moreover, dietary supplementation of 0.1% 10-HDA had comparable or even better protection for the LPS-challenged chickens than supplementation with antibiotics or 0.5% 10-HDA. These results suggest that 10-HDA has the potential to be used as an alternative to antibiotics in protecting the intestinal health and improving the performance of poultry.

Gut microbiota can promote the onset and acquiring of the early development of the passive immunity, improve immune tolerance, and maintain normal communications between the gut epithelia in the newborn neonates. Peng et al. demonstrated that early flora colonization affected the gut epithelial apical expression of the Fc receptor (FcRn) and the FcRn-mediated intestinal IgG uptake in the neonatal piglets, which may be mediated by the NF-κB-FcRn pathway. Milk replacers have been shown to increase the diversity and richness of the microflora in the calf ileum (Wang Y. et al., 2021). The results by Badman et al. (2019) showed that the differences in the nutritional composition of bovine milk replacers had a major impact on microbiota composition, diversity, and succession in pre-weaned dairy calves, further influencing the health of the gut and the whole animal. The effects of milk, milk replacer and ethoxyquinoline on growth performance, weaning stress and fecal microbiota were evaluated in dairy cows; and it was suggested that milk replacer plus ethoxyquin enhanced the defensive capacity and improved microbial composition in mitigating the negative effects of weaning in calves (Wei et al.). The effects of yeast polysaccharide (YPS) supplementation on lactation and growth were studied in Texas donkeys, burros, and foals and it was found that dietary supplementation of YPS enhanced feed intake, boosts immunity by increasing immunoglobulin levels, stimulates the growth-promoting gut microbiota (e.g., *Lactobacillus and Prevotella*), and exerted no adverse effects on performances of both Dezhou donkey jennies and foals (Huang B. et al.).

The gut microbiota is essential for extracting energy and other dietary macro-nutrients from plant foods and may help animals adapt to new dietary environments in response to rapid environmental changes especially in wild life species. Sun et al. analyzed the differences in the composition, structure and function of the intestinal microbial community of the François langur (*Trachypithecus francoisi*) under different environments and diets by using metagenomic sequencing technology. Their results showed that the structure, composition, diversity and function of the intestinal microbial community of *Trachypithecus francoisi* varied with different environments and diets (Sun et al.).

The effects of dietary adding oregano essential oil (OEO) on intestinal barrier integrity, colonic microbiome composition and microbial metabolite production were investigated in fattening bulls and the results showed that OEO facilitated intestinal luminal environmental homeostasis by promoting the growth of beneficial bacteria while inhibiting harmful ones (Ma et al.). Dietary addition of Prickly ash seeds (PAS) increased the diversity and abundance of intestinal microorganisms in Hu sheep, influenced the composition of the intestinal community in the sheep, increased the content of beneficial intestinal bacteria, and influenced the level of inflammatory factors, thereby improving the immunity of the sheep lambs (Li et al.). In another study for evaluating the potential regulatory effects of *Allium mongolicum* regel ethanol extract (AME) on *in vitro* ruminal fermenting biohydrogenation (BH) bacteria, a significant reduction in the abundance of rumen BH microflora was observed by AME (Wang X. et al.)

Tens of thousands of microflora grow together in the gut with their hosts to form a complex and variable microecosystem that plays an important role in host nutrition, growth, metabolism and immunity. Changes in the microflora allow the host to rapidly adjust its metabolic and immune properties in response to environmental changes. Spermidine can regulate a variety of biological processes and plays a crucial role in maintaining cellular homeostasis, regulating immune function, promoting cell proliferation and antioxidant function (Wang Z. et al.). Concentrations of spermidine are high in plant-derived foods, especially in wheat germ and other feed ingredients (Wang Z. et al.). Spermidine is a suitable therapeutic for clinical applications, as it has low biotoxicity and is highly effective at low to moderate concentrations (Wang Z. et al.). Studies have shown that exogenous supplemental spermidine increases the abundance of *Fournierella* and *Anaerofilum* flora in the intestinal tract of calves (Wang Z. et al.). Spermidine was found to improve gut health by improving intestinal morphology, increasing antioxidant capacity, and modulating the structure of the intestinal flora in the Sichuan white geese (Wang Z. et al.). Another study showed that peppermint extract improved egg production and quality, increases antioxidant capacity, and alters cecal microbiota in late-phase laying hens (Bai et al.). Good intestinal structure and function are the basis for a variety of life activities in poultry and livestock. The intestinal tissue is the largest second immune organ of the organism and the interface between the organism and the outside world. The proliferative use of antibiotics and intensive farming patterns have led to more serious intestinal health problems in poultry and livestock production, and the intestinal tissues are susceptible to external factors such as feed, microbial metabolites and temperature, leading to oxidative stress and reduced health levels. The level of intestinal health is mainly related to intestinal mucosal integrity and structure, antioxidant capacity and flora structure. Improving the intestinal health of poultry and livestock has become an important way to resolve the health problems of food animal production in “resistance-free” farming.

The composition and diversity of herbivore gut microorganisms are also influenced by dietary bioactive compounds. The effects of kinnikinnick tannins (CKT) on fermentation, methane emissions, methanogenic bacterial communities, and ruminal metabolome were studied in sheep; and CKT supplementation was found to lead to a reduction in daily CH_4_ emissions from sheep by decreasing the abundance and diversity of ruminal methanogenic bacterial communities, while simultaneously decreasing tyramine concentrations and increasing N-methyl-L-glutamic acid concentrations (Niu et al.). The effects of American ginseng pomace as a potential feed additive on the production performance and the gastrointestinal bacterial community responses were comprehensively evaluated in antlered sika deer (Wu et al.). Their results showed that dietary addition of the Western ginseng pomace increased the apparent digestibility of nutrients, improved immune and antioxidant status, facilitated antler production in antlered Merganser during the antler-antler stage, and positively regulated gastrointestinal flora and bacterial fermentation (Wu et al.).

Three studies in this special Edition investigated how diets affected gut health, nutrient utilization and growth performances in pigs. Effects of dietary supplementation of pharmacological level of zine oxide (ZnO) and condensed tannins (CT), independently or in combination, on the growth performance and intestinal health of weaned piglets in the enterotoxigenic *Escherichia coli* (ETEC-K88)-challenged environment were evaluated (Yi et al.). It was found that ZnO increased the ileum villus height and improved intestinal barrier function by increasing the content of mucin 2 (MUC-2) in jejunum and ileum mucosa and the mRNA expression of zonula occludens-1 (ZO-1) in jejunum and the expression of Occludin in duodenum and ileum (Yi et al.). The effects of CT on intestinal barrier function genes were similar to that of ZnO; the mRNA expression of cystic fibrosis transmembrane conductance regulator (CFTR) in jejunum and ileum was reduced in the ZnO group; and CT was also capable of alleviating diarrhea by decreasing CFTR expression and promote water reabsorption by increasing AQP3 expression (Yi et al.). In addition, pigs receiving ZnO diet had higher abundances of *Bacteroidetes* and *Prevotella*, and lower *Firmicutes* and *Lactobacillus* in the colonic contents (Yi et al.). These results indicated that dietary ZnO and CT can alleviate diarrhea and improve intestinal barrier function of weaned pigs in an ETEC-challenged environment. Sarpong et al. revealed that gut microbial signatures and enterotype clusters were implicated in the efficiency of nitrogen utilization in fattening pigs. It was demonstrated that dietary supplementation of dried whole-plant silage maize could improve fiber digestive characteristics via modulating intestinal microbiota in the indigenous Hezuo pig (Wang L. et al.).

The interplay between gut microbiota and immunity is relatively obscure and complex. First, the gut microbiota can recognize nutrients and antigens by inducing the immune system to tolerate commensal bacteria. On the other hand, gut microbiota can also prevent bacterial invasion and infection through immune recognition. Modern studies have shown that gut microbiota and body immunity are interdependent. In some patients with autoimmune diseases, the diversity and abundance of gut microbiota are severely disrupted (Zhu et al.). Numerous other factors that may modulate the gut microbiome, such as diet, medications, mental health, and environmental factors, are noteworthy for various diseases such as tumor and cancer treatments. Ongoing published studies have provided interesting results for the scientific and global community, and to conclude, considerable progress has been made over the past two decades. From initial controlled animal studies and clinical observations to more mechanistic approaches, the field of gut microbiota in animal and human nutrition and health is evolving into one of irrefutable causation. However, there are still many studies claiming causality when in reality only correlation has been demonstrated. The shift from correlation to causation remains an important and necessary step to better design hypothesized interventions based on modulation of the gut microbiota or the use of specific dietary active compounds. The scientific community is moving toward personalized food for medicine and health due to advances in many aspects of research and innovation, and the gut microbiome era is clearly an important part of the future paradigm shift in the nutritional and medical application approaches.
